# Psychological and Physiological Stress in Hens With Bone Damage

**DOI:** 10.3389/fvets.2020.589274

**Published:** 2020-12-15

**Authors:** Neža Rokavec, Manja Zupan Šemrov

**Affiliations:** Department of Animal Science, Biotechnical Faculty, University of Ljubljana, Domžale, Slovenia

**Keywords:** keel bone, poultry, stress physiology, behavior, body development, affective state

## Abstract

Abnormalities in bone development in humans and non-humans can lead to impaired physical and psychological health; however, evidence is lacking regarding the role of individual psychosocial factors in the development of poor bone conditions. Addressing this lack of knowledge, we used low-productive laying hens (*n* = 93) and assessed behavioral responses to an open-field test [at 17, 18, 29, 33 weeks of age (wa)], an aerial predator test (at 39 wa), and a social reinstatement test (at 42 wa). Bone condition was assessed using a palpation technique on five occasions (at 16, 29, 33, 45, 58 wa), with half of the hens experiencing damage (deviations, fractures, or both) at 29 wa and all hens by 58 wa. Corticosterone (CORT) concentration in feathers (at 16, 33, 58 wa) and body weight (at 23, 47, 58 wa) were also investigated. We hypothesized that lighter birds (at 23 wa) with higher CORT (at 16 wa) and open field-induced fear collected before the onset of lay (at 17 and 18 wa) are associated with a worse bone condition when in lay. We also hypothesized that those birds with more damage at the peak of laying (at 29 wa) would be lighter at 47 and 58 wa and more fearful by showing higher open field-induced (at 29 and 33 wa) and predator-induced fear responses, however, acting less socially toward conspecifics. These hens were also expected to have higher CORT (at 33 and 58 wa). Our results show no association between open-field fear level and fear behavior, CORT concentration, or body weight on the one hand (all measured before starting to lay) and bone damage at 29 wa on the other. When in lay, bone damage was associated with more pecking and less crossing zones when faced with an open-field situation at 29 wa and improved sociality at 42 wa. This study provides the first evidence of a relationship of bone health with fear, sociality, and stress response. When in poor bone condition, our hens had enhanced psychological stress measured by fear behavior reactivity but not physiological stress measured as feather CORT concentration.

## Introduction

Bone disease, such as osteoporosis in humans, is often seen as a silent disorder until it causes fractures (1). Yet, the consequence of such disease can have a major impact on individuals such as a decrease in physical and psychological health. Many humans who suffer from bone fractures experience significant pain and weight loss; they may lose the ability to stand and walk (2) or may be immobilized by a fear of falling (3) or even begin to feel isolated and helpless (2). On top of these effects, an increase in indirect costs [e.g., lost productivity for patients and caregivers (2) and increased stress level (4)] has recognized.

Animal welfare scientists agree that laying hens suffer from a variety of welfare problems, including the keel bone damage (KBD) (5), which is estimated to reach a prevalence of between 30 and 90% by 45 weeks of age (wa) when the ossification of the keel bone (KB) is completed (6–8). KBD includes both fractures and deviations (9). Unlike fractures, which usually happen during an isolated event such as crashes/collisions during flight or uncontrolled landings and takeoffs (9), the development of deviations happens over a period of time as an outcome of bone remodeling in response to regular loading pressure during roosting (7). Bone damage is known to affect a broad spectrum of issues in the poultry industry, including egg production (8, 10, 11), water intake (12), body weight (6), deformation of breast muscle (12), and to cause welfare problems (9) including pain (13). In 1868, Darwin became the first to document (14) that egg-producing domestic fowl, laying fewer eggs than the hens nowadays, exhibited KBs that were moderately crooked or extremely deformed. In the early 1990s, crooked keels in laying hens were ascribed to hereditary disease (15), rickets (16), faulty metabolism, or a slow process of ossification (17). Despite this, only recently has research intensively focused on looking at the underlying causes and consequences of KBD in commercial laying hens.

Recent findings (10, 12, 18) reveal that it is likely that production is just one of many factors affecting bone integrity, explaining why some studies found no effect on egg production (6, 12) or body weight (19). By reviewing several research studies, Riber et al. (9) summarized that psychological stress factors may be related to bone damage and that KBD promotes the expression of negative states. In layers, investigating negative affective states focused mainly on pain (13, 19) or the fear level (20–22), often related to fear of humans (22), while for positive states, it concentrated on assessing social behavior (21). Studies also found that bone damage affects not only welfare but physiological parameters (10), although the problem's multifactorial nature makes it difficult to study the underlying causes and consequences of KBD in commercial laying hens.

To date, insufficient longitudinal data have been available to link bone damage and emotional consequences or to investigate the hypothesis that the affective state may impact damage. Riber and Hinrichsen (23) suggested, albeit have not yet clearly demonstrated, that a link exists between injurious pecking damage, bone damage, and fearfulness. Recently, by investigating changes in the hippocampus in a small number of commercial Lohmann Brown hens (15 hens with severe and nine hens with minimal KB fractures) in an aviary system, Armstrong et al. (24) found that hens with KB fractures are more likely to experience negative affective states that last for at least 3–4 weeks. In line with this and the fact that fearfulness of an individual, which is a known measure of psychological stress and thus, a negative affective state, could affect physical health (25) and its sensitivity to physiological stress (26), our main objective was to investigate the relationships between the affective state recorded during behavioral testing and the development of bone condition, corticosterone (CORT) concentration, and body weight. Levels of CORT deposited in feathers were analyzed to provide a measure of longer term physiological stress [i.e., (26, 27)]. Slovenian locally adapted laying hens of the Styrian breed (n = 93) were subjected to standardized test situations [i.e., open-field test (OFT), aerial predator test (APT), social reinstatement test (SRT)], and the level of fear and sociality were measured. We chose this particular strain of bird to improve our understanding of the behavioral and stress responses of hens with low egg production and good resistance to diseases (28); their bone condition is expected to be less likely poor and to show greater variation in behavioral responses compared to highly productive hens that have been intensively selected. Moreover, thus far, there are no data on the association between the prevalence of KBD in non-commercial chicken breeds and affective states. Assuming lighter birds are more prone to show fear behavior and be fearful (29), fearful birds have a higher risk of injuries (30), and bone development depends on the concentration of glucocorticoids in humans (2) and animals (18, 31), we first hypothesized that lighter individuals that show more fear-related characteristics and a higher stress-induced CORT before starting to lay will have a poorer bone condition at a later time point and, second, that those birds with more damage at the peak of laying will show more fear but act less socially toward conspecifics and have a lower body weight and a higher CORT. The latest hypothesis was derived from human studies (2–4) but also from the suggestion that when small prey animals are subjected to fear stimuli such as predator-like stimulus, this may elevate long-term stress and defensive responses and may lead to future stress-induced weight loss (32).

## Materials and Methods

### Animals and Housing

The experiment was conducted from October 2017 to August 2018 at the Krumperk Educational and Research Centre, University of Ljubljana, Biotechnical Faculty. Randomly selected pullets (*n* = 93) and cockerels (*n* = 15) were obtained from a commercial flock of a basic floor-rearing system at 16 wa and transported to the laying pen (l × w = 865 × 496 cm). From 16 to 58 wa, the flock was kept in this barn system with wood shavings (7-cm depth) and started to lay at 23 wa. To allow recognition, all females were marked with leg rings. The laying pen was divided by a wire mesh into a smaller (l × w = 865 × 186 cm) and a larger (l × w = 865 × 310 cm) area linked by an always-open door. Light was provided by two bulbs according to a 14:10 h light:dark cycle. Chickens had free access to a standard commercial layer diet from three round feeders (at 27 cm height) and water from drinking lines (at 37 cm height) with 25 water nipples in the smaller area and 29 in the larger area. Three wooden perches were placed in the middle of the larger area, each with dimensions of 190 × 4 × 6 cm, placed at a height of 66 cm above the ground. The pen contained two metal nest box lines at a height of 50 cm above the ground when measured from the lower line, with 14 nest boxes each (w × d × h = 30 × 30 × 30 cm) and three wooden perches (l × w × h = 200 × 4 × 2 cm) in front. The available perch space was 12.9 cm per bird. Two automatic axial propeller fans were used to draw air out of the building through the wall vents (negative pressure ventilation), and two air inlets were used to ensure fresh air entered the barn.

### Experimental Design

**Figure 1:**
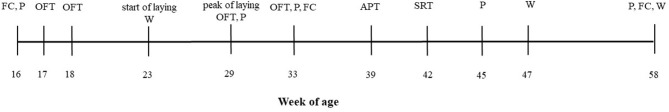
The timeline of the measurements taken. FC, feather collection; P, palpation; W, body weight; OFT, open-field test; APT, aerial predator test; SRT, social reinstatement test.

Figure 1 illustrates the different experimental procedures carried out over a period of 42 weeks, during which the hens were individually weighed (at 23, 47, 58 wa), exposed to three different behavioral tests, and palpated to record KB status. To obtain a retrospective measure of the long-term stress experienced by the birds during feather growth (27, 33), feathers were taken at three time points for analysis of CORT concentrations. These procedures are explained in more detail in the following sections and were chosen to investigate the relationships between fear as an indicator of psychological stress, CORT as an indicator of physiological stress, and KBD as well as body weight as a physical condition.

### Palpation of the Keel Bone

The presence of both fractures and deviations of the KB was assessed by palpation on five occasions (at 16, 29, 33, 45, and 58 wa) using the Simplified Keel Assessment Protocol (SKAP) palpation system (34). At 16 and 29 wa, only the presence or absence of damage was recorded, whereas at 33, 45, and 58 wa, the type of damage (deviation, fracture, or both) was also specified. The person assessing the damage was trained on how to palpate hens during a 2-day course at the University of Bern in 2017. The study of this training school revealed that training with radiographs improved palpation accuracy (35). On each assessment occasion, hens were taken from the pen in random order and transferred to a nearby room. Each hen was held in the observer's arms in the position of a cradle and the ventral and lateral surfaces of the KB were palpated by running the forefinger and thumb up and down the bone.

### Feather Collection and Corticosterone Analyses

Feather collection was done by cutting the primary third feather of the wing from each hen. The first feather was representative of the period before the onset of lay and cut out at 16 wa. The second feather was cut at 33 wa, i.e., 4 weeks after the peak of lay, and the final one at the end of the experiment (58 wa). The first and the third feathers (that grew out between 16 and 58 wa) were taken from the left wing and the second feather from the right wing. Feathers were stored separately in a paper envelope and kept on a shelf at ambient indoor temperature before analysis. A methanol-based extraction technique was used to extract CORT from feathers [adjusted after Bortolotti et al. (27)]. The feathers were prepared by cutting vanes into pieces with scissors. From each feather, 30 mg was used and put into a test tube. Then, 10 ml of methanol gradient grade for liquid chromatography (Merck KGaA, Darmstadt, Germany) was added, and the samples were placed in a sonicating water bath at room temperature for 30 min, followed by incubation at 50°C overnight in a shaking water bath. The methanol was then separated from the feather material by basic gravity filtration using a cellulose filter paper in the filtration funnel. The feather remnants, original sample vial, and filtration material were washed twice with ~2.5 ml of additional methanol; the washes were added to the original methanol extract. The methanol extract was placed in a 50°C water bath and subsequently evaporated in a fume hood under nitrogen gas. Evaporation of the samples was completed within a few hours, and the extract residues were reconstituted in 500 μl of 15% methanol. Reconstituted samples were frozen at−20°C until analyzed for CORT. The concentration of the samples (nmol/L extract) was assayed using the commercially available ELISA kit (DE4164, Kiel, Germany). The test procedure followed standard methods. While calculating the CORT concentration in feathers (pM/g), the dilution factor (16.67) was taken into account. The CORT concentration represented three measures (CORT at 16 wa represented the storage between 0 and 16 wa; CORT at 33 wa represented the storage between 0 and 33 wa; CORT at 58 wa was the sum of CORT at 16 wa and CORT at 33 wa).

### Behavioral Tests

Starting at 17 wa, the hens were subjected to several behavioral tests. The tests were performed in the same order for each hen, i.e., first the OFTs, then APT, and finally the SRT. The OFT was performed four times at different ages in order to investigate intra-situation coping responses. As for inter-situation behavioral responses that may represent generalized fearfulness, APT and SRT were performed at later ages but were not tested in weeks in which palpation or feather collection were carried out in order to avoid confounding the behavioral readouts. Consistency of behaviors in different tests was investigated. Hens were individually caught from the pen, each time from a different location in order to avoid biases in the test order as less fearful or slower birds are often picked first, and brought to the test apparatus in one person's hands. Their behaviors were recorded using direct observations by one observer. Recorded by stopwatch, timing started 30 s after being placed in the test apparatus.

#### Test Apparatus

**Figure 2:**
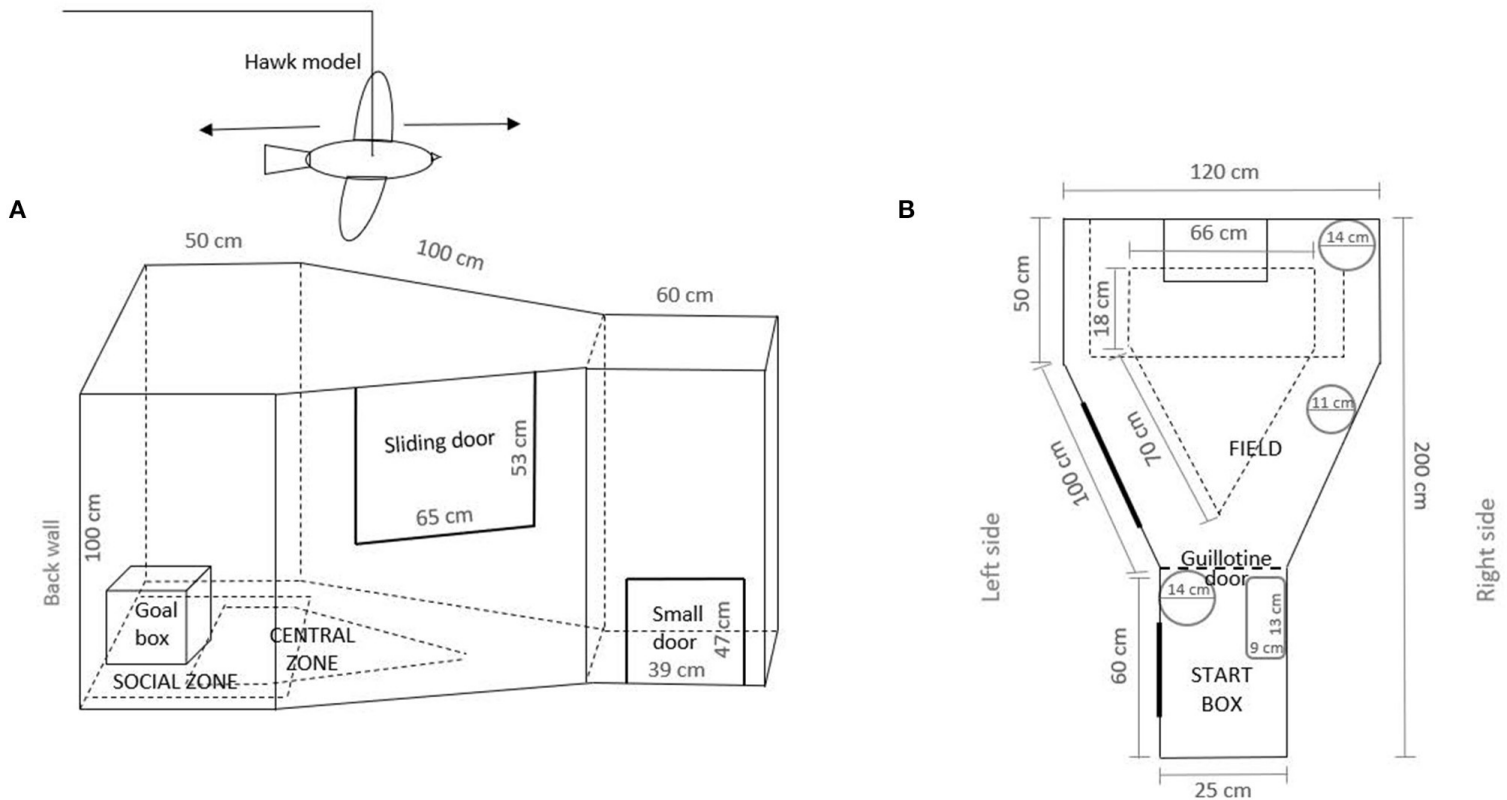
Experimental apparatus showing **(A)** side view and **(B)** top view.

The apparatus (Figure 2) was a weather-resistant black plywood (T-fix) trapezoid-like arena isolated from humans and animals [adjusted after de Haas et al. (36)]. It was located in a 474 (l) × 360 (w) × 258 cm (h) room next to the laying pen and illuminated with a light bulb of 206.9 lx (measured at the hen's head). An extra light was placed 140 cm above the apparatus to help track the test hen and ensure visibility. A hen was placed in the start box through a small door and then introduced into the field arena when the guillotine door of the start box was opened. Observations started when the guillotine door was opened. To measure movement, the arena was divided into zones (i.e., central and social zone) marked with black tape. A human could gain access through the sliding door to catch a bird and to clean (i.e., vacuuming feathers and wood shavings and absorbing feces with cellulose) the area after each testing. By using mirrors, the observer standing behind the start box was out of view of the test subjects so as not to influence their behavior. The circular and rectangular mirrors were placed above the guillotine door of the start box. Other circular mirrors were on the back wall and on the right side of the apparatus. Part of the scoring criteria included flying out of the arena, which is why the top of the arena was not covered.

#### Open-Field Test

The hens (*n* = 93) were individually exposed to an OFT at 17, 18, 29, and 33 wa between 9:00 and 15:00 h. Each of them lasted 3 min. At 10, 20, 30, 40, 50, and 60 s after opening the guillotine door, the birds' fear responses were categorized as calm, ambiguous, fearful, and highly fearful according to specific behaviors detailed in predetermined selection criteria (Table 1), and the fear scores were averaged across the six observations.

**Table 1:** Selection criteria for fear responses in the open field test.

**Fear response**	**Behavior**	**Fear score**
Calm animal	Exploring, standing or walking, short or normal length of the neck, and no vocalizing or vocalizing quietly (calm, low)	20
Ambiguous animal	Standing or walking, neck stretched, head flicks, and no vocalizing or vocalizing quietly (calm, low)	40
Fearful animal	Standing or walking, neck stretched, head flicks, and vocalizing loudly	60
Highly fearful animal	Escape, attempting to escape, and vocalizing loudly or no vocalizing. The bird is completely still (freeze behavior).	80

*Fear scores were adapted from Agnvall et al. (37). Fear responses and behaviors were newly defined*.

Other fear behaviors (Table 2) were recorded for 3 min of testing using continuous sampling. Pecking and preening were added because they frequently occurred during the pilot study.

**Table 2:** Ethogram of the behaviors recorded during the tests and their descriptions.

**Test**	**Behavior**	**Description**
**OFT**		
	*Latency to leave the start box*^*D*^	Length of time from the start of testing to stepping in the field with both feet
	*Latency to reach the central zone*^*D*^	Length of time when both feet reach into the central zone
	*Crossing the central zone*^*F*^	Defines how often the hen crosses the central zone
	*Preening*^*F*^	Defines how often the hen moves its head in a smoothing motion over the body
	*Pecking*^*F*^	Defines how often the hen pecks on the ground or at the wall of the platform as visual inspection
**APT**		
	*Activity*^*F*^	Defines how often the hen has a relaxed body stance, short or normal length of the neck (when she moves, stands, or sits) and does not vocalize or vocalizes quietly
	*Freeze*^*F*^	Defines how often the hen is completely still*
	*Escape attempt*^*F*^	Defines how often the hen tries to escape, i.e., constantly looks up at the top of the platform with neck stretched or tries to fly out
	*Being alert*^*F*^	Defines how often the hen has an alert body stance with neck stretched (when she moves, stands, or sits) and does not vocalize or vocalizes loudly
	*Latency to escape*^*D*^	Length of time from the start of testing to the platform breakout
**SRT**		
	Vocalization^*F*^	Defines how often the hen vocalizes
	*Latency to vocalize*^*D*^	Length of time to the first sound the hen makes
	*Latency to escape*^*D*^	Length of time from the start of testing to the platform breakout
	*Latency to leave the start box*^*D*^	Length of time from the start of testing to stepping in the field with both feet
	*Latency to reach the social zone*^*D*^	Length of time when at least one of the feet reaches the social zone or a hen jumps from the field on the cage located in the social zone
	*Duration in social zone*^*D*^	Time spent in the social zone*

F *behavior recorded as frequency*;

D behavior recorded as duration; OFT, open-field test; APT, aerial predator test; SRT, social reinstatement test. *descriptions were adjusted after Agnvall et al. (37).

#### Aerial Predator Test

Behavior was observed during a simulated aerial predator attack that was carried out once at 39 wa, between 9:00 and 14:00 h, to investigate the initial response to a potential natural predator. For behaviors (Table 2), we used instantaneous sampling with 10-s intervals during 2 min of testing, while continuous sampling was used for latency to escape. In order to obtain a baseline of the behavior, the animals were first observed undisturbed. After 1 min of opening the guillotine door, a hawk-silhouette model (Figure 2) (measuring 41.0 × 22.4 cm) made out of brown-colored plywood (natural color of the falcon) was pulled back and forth along a string starting 140 cm above the testing room floor, 15 cm from the back wall of the apparatus. The model passed through the arena's 340 cm in 3 s. Before and after the simulated overflight, the hawk silhouette was hidden behind a gray curtain.

#### Social Reinstatement Test

At 42 wa, the hens' level of sociality (motivation to be with conspecifics) was measured between 8:00 and 12:00 h. Beside the back wall of the arena, one stimulus hen familiar to the test bird (one of 93 test hens) and of the same age, was kept in a wooden framed box (Figure 2) of 30 (l) × 40 (d) × 40 (h) made of wire mesh. The stimulus bird was changed after each test. An area close to the social companion was defined as a social zone, marked with black tape at 25 cm around the goal box. This distance was chosen according to Dawkins (38), who claims that social recognition in hens may only occur at distances <30 cm. The hens were tested once. The test procedure was as follows: when the test started, the behaviors described in Table 2 were observed for 3 min using continuous sampling.

### Statistical Analysis

Five hens unexpectedly and unrelated to the experiment died before 58 wa and were excluded from certain analyses. Further, feather CORT concentration was missing for four hens at 16 wa and for one hen at 33 wa. The statistical analysis was performed using the SAS/STAT software, version 9.4, of the SAS System for Windows © 2002–2012 SAS Institute Inc. The normal distribution for quantitative traits was determined by the Shapiro–Wilk test. All reported *P* ≤ 0.05 were classified as statistically significant while <0.06 as with a strong tendency.

Considering the behavioral observations, latencies to leave the start box and to reach the central zone among repetitions of OFT were analyzed in a survival analysis context (39) by the PHREG procedure, where non-events were treated as right-censored data. Preening and pecking as well as APT behaviors were calculated first as frequencies per hen and then as number of times per time period for all the tested birds. A difference between the behaviors observed in OFTs, as well as before and after a hawk appearance in APT, was investigated using the chi-square test. Consistency was computed for behaviors in repeated OFTs as suggested by Nakagawa and Schielzeth (40), where behaviors were treated as binary variables with 1 (i.e., event) and 0 (i.e., non-event). A multinomial overdispersion model was used in the GLIMMIX procedure, and consistencies are presented on a latent scale.

In the following step, the KBD as a binary outcome (1, damage; 0, no damage) and CORT concentration as a continuous variable at different ages were analyzed by the FREQ procedure using the CMH option in the TABLES statement or by the MIXED procedure with a hen treated as a random effect, respectively. Since the majority of hens experienced KBD at 33 wa (80.6%) and all hens at 58 wa (100.0%), it was only possible to use KBD data at the peak of lay (29 wa), where 50% of hens were found with KBD for further analysis. Using the LOGISTIC procedure with modeling probability that KBD is 1, we investigated if the average fear score at 17 (OFT1) and 18 wa (OFT2) and fear-related behavior responses in OFT1 and OFT2, body weight at 23 wa, and CORT at 16 wa affected KBD. We were further interested to know if the presence/absence of KBD was related to the average fear score at 29 (OFT3) and 33 wa (OFT4), body weight at 47 and 58 wa, CORT at 33 and 58 wa, and the behavior responses displayed in OFT, APT, and SRT. In the original models, all of the tested variables were included, but those found not significant for KBD were removed. Wald chi square statistics was provided for results deriving from the LOGISTIC procedure.

## Results

### Behavioral Tests

#### Open Field Test

With repetitive exposure to the OFT, more hens left the start box (χ^2^ = 25.43, df = 4, *P* < 0.0001) and did so faster (χ^2^ = 72.02, *P* < 0.0001; data not shown). The highest number of hens reached the central zone in OFT3 (χ^2^ = 17.75, df = 3, *P* = 0.0005), and they did so more frequently in OFT3 and OFT4 than in OFT1 and OFT2 (χ^2^ = 40.62, df = 3, *P* < 0.0001; data not shown). There were six hens that did not leave the start box in any of the four repetitions. The frequencies of preening (from OFT1 to OFT4 test; 1: *n* = 57, 2: *n* = 104, 3: *n* = 72, 4: *n* = 62; χ^2^ = 18.13, df = 3, *P* = 0.0004) and pecking (from OFT1 to OFT4 test; 1: *n* = 104, 2: *n* = 150, 3: *n* = 127, 4: *n* = 145; χ^2^ = 9.89, df = 3, *P* = 0.02) also differed among repetitions.

Consistency of behaviors treated as binary traits in repeated OFTs was low to moderate. Leaving the start box over four repetitions of OFT had consistency of 0.181, and with inclusion of SRT, it increased to 0.188. If only records from the rearing period were considered, consistency was 0.211, and for records from only the laying period, it was 0.561. Consistency for reaching the central zone was lower than consistency for leaving the start box, with higher values in the rearing and laying period separately compared to the inclusion of all four repetitions of OFT. Preening had the highest consistency of the behaviors observed; 0.345 for four repetitions of OFT, 0.442 in the rearing and 0.493 in the laying period. Consistency for pecking was 0.159 for four repetitions of OFT, 0.093 in the rearing and 0.289 in the laying period.

#### Aerial Predator Test

The hens' behavior differed when comparing responses before and after the appearance of the hawk. More exploring before its appearance (283 vs. 172 times; χ^2^ = 27.08, df = 1, *P* < 0.0001) and less standing alert (139 vs. 202 times; χ^2^ = 11.64, df = 1, *P* = 0.0006) were observed. No differences were observed in freezing behavior (89 times before vs. 111 times after; χ^2^ = 2.42, df = 1, *P* = 0.12) and escape attempts (31 times before vs. 32 times after; χ^2^ = 0.02, df = 1, *P* = 0.90). Some hens froze (before: *n* = 22; after: *n* = 27), and others tried to escape the test apparatus (*n* = 18), but few managed to escape (before: *n* = 5; after: *n* = 3).

#### Social Reinstatement Test

In the SRT, hens (*n* = 78) left the start box in 23.85 ± 3.30 s, 53 hens reached the social zone in 63.21 ± 6.12 s and stayed in the zone for 100.75 ± 6.98 s. They needed 55.42 ± 7.07 s to start vocalizing (*n* = 48 hens), with the maximum number of events per hen being 18. Some hens (*n* = 10) escaped from the test apparatus with a latency of 49 ± 14.79 s.

### Keel Bone Damage

The number of hens exhibiting bone damage (deviations and fractures combined) increased with age (Mantel–Haenszel χ^2^ = 21.86, df = 1, *P* < 0.0001), with most hens without KBD at 16 wa (6.5%), half of the hens with KBD at 29 wa (50.4%), almost all hens at 45 wa (94.6%), and all hens showing KBD at 58 wa (100%). CORT concentration was also found to increase with age (mean ± SD; 16 wa = 44.78 ± 16.07; 33 wa = 67.83 ± 29.73; 58 wa = 96.95 ± 25.03; *F* = 696.81, df = 2, *P* < 0.0001). Hens weighed 1435.05 ± 164.11 g at 23 wa, 1795.22 ± 213.24 g at 47 wa, and 1825.39 ± 226.33 g at 58 wa.

Before starting to lay and by using a logistic regression model, we found that body weight affected KBD by lighter hens showing more bone damage (Wald χ^2^ = 4.65, *P* = 0.03), but biological significance was negligible (for a 1 kg heavier hen probability increased by only 0.3%). CORT stored from 0 to 16 wa had no relationship to KBD at 29 wa (Wald χ^2^ = 0.17, *P* = 0.68) nor had the average fear score at 17 (Wald χ^2^ = 1.28, *P* = 0.26) or 18 wa (Wald χ^2^ = 0.49, *P* = 0.48). When in lay, pecking and frequency of crossing zones at 29 wa in OFT3 as well as latency to leave starting arena in SRT showed a relationship with KBD (Table 3). Hens with bone damage at 29 wa were pecking more but crossing zones less in the OFT and reaching the testing arena faster in SRT. No other relationships were found including those between KBD and average fear scores (data not shown).

**Table 3:** Behavior responses as frequency (mean ± SE) associated with KBD at 29 week of age (wa) in OFT and SRT.

	**With KBD**	**Without KBD**	**Wald**	***P***
			**Chi-Square**	
Pecking^OFT^	1.64 ± 0.33	1.09 ± 0.24	3.78	0.052
Crossing the central zone ^OFT^	1.13 ± 0.18	1.41 ± 0.21	5.81	0.02
LLAS (s)^SRT^	17.73 ± 3.41	31.76 ± 5.94	6.62	0.01

*KBD, keel bone damage; OFT, open-field test; SRT, social reinstatement test; LLAS, latency to leave the start box*.

## Discussion

This study used fowl as an animal model to investigate the relationship between fear and stress responses and bone health. We found multiple relationships. Although we cannot confirm that the patterns we observed in the predator- and open field-induced fear situations are individual behavioral strategies stable over a longer time, our results support the existence of a relationship between psychological stress experienced as fear and the development of physical health reflected in bone condition. In contrast, we were unable to confirm that bone condition was associated with either physiological stress (measured as feather CORT concentration) or body weight.

In the OFT, the individual behavior was determined by different quantities of fear behavior (as number of times the target behavior was observed; Table 2) and was inconsistent with time, however, associated with bone condition at the peak of laying period (at 29 wa). This means that an individual used a strategy on an *ad hoc* basis based on how good/bad its keel health was. The behavior displayed seems to depend also on an individual life stage need (41) or may result from an increased willingness to move from the start box or from a habituation effect (42, 43), since being in lay led to an increase in the number of our hens leaving the start box and reaching the central zone. This change might also depend on age or early experience because fearful shyness occurs among the young of most mammalian species (44), which could also be true for the bird. Considering the impact of affective states on bone damage, our results show that fear responses categorized from calm to highly fearful, a high psychological state of fear at 17 and 18 wa, but also high physiological stress assessed by feather CORT concentration (at 33 and 58 wa) or low body weight (at 47 and 58 wa) were not associated with less bone damage at 29 wa, which is contrary to our expectations. As reviewed by Harlander-Matauschek et al. (30), it could be that with regard to KB fractures, fearful hens may be more likely to panic and thus collide with pen furnishing, leading them to develop worse bone health. Our current results are incongruent with expectations based on this literature. However, no study has been reported to clearly investigate and demonstrate the link between underlying fearfulness and bone damage in animals. Still, one possible reason for not confirming our first hypothesis is the exposure of our hens to only two acute fear-induced situations before the bone damage reached the prevalence of half of the hens. One could also argue that the responses recorded are unrepresentative of actual fearfulness because they were not investigated in the hen's home pen.

Considering the consequences of bone damage, hens with bone damage at the peak of the laying period (29 wa) had similar feather CORT concentrations at 33 and 58 wa and body weight at 47 and 58 wa. When exposed to open field-induced fear situation at 29 wa, these hens were recorded as having moved less often between zones in the test arena. According to the assumptions of the OFT (45), this can be a measure of worse locomotor behavior in chickens. Since these hens also showed more pecking on the ground and the wall, it is less likely that this behavior is a sign of exploratory pecking. Given the presence of escape attempts (recorded as fear responses and labeled as highly fearful animals), these responses suggest that they perceived the situation as more threatening or fearful. These findings also suggest that fearfulness, bone damage, and pecking behavior are related, a link proposed previously with injurious pecking behavior (23).

In many species, fear level has been negatively correlated with social motivation [birds (46), pigs (47), horses (48)], and this has been linked to high physiological stress responses [birds (49), pigs (50), humans (4)]; however, our results contradict these links. In the social situation at 42 wa, the responses of hens with bone damage suggest improved sociality with animals leaving the start box. According to models of motivation (51), these responses may indicate that individuals had a higher motivation to explore a subject/environment or a lower fear or anxiety level in a social context. However, this result may also be interpreted as a sign of fast decision-making (52) or boldness (53) with active seeking to escape and social reinstatement (54) or sensitization (55). When coupled with the argument of Mills et al. (46) that social motivation predominates over the fear response in individuals with a high tendency of making social contact, hens with bone damage imply to develop a different biological sensitivity to the social context (44) compared to hens without damage. Whatever the reason, it appears that bone damage, which potentially causes pain, particularly when bone is broken (5, 9), leads to different fear- and social-related psychological stress in birds, but not necessarily stress-induced CORT. It must be emphasized that this interpretation is based on an analysis where fractures and deviations were considered in a single variable due to the method applied, which is most practical from a commercial perspective but is not reliable enough to detect all differences in damage nor the time of a fracture. Evidence suggests that bone fractures have a negative impact on self-esteem, body image, and mood in humans (2) as well as negative affective states in laying hens (24).

It has also been documented that humans (2, 56) and animals [dogs (57), chicken (58), mice (59)] can encounter problems with bone condition due to body weight. It remains unclear why in species, such as birds, the KB is not under the influence of body weight, although it is in conjunction with the studies of Nasr et al. (13, 19) using a highly productive Lohmann Brown laying strain. One possible explanation for not detecting its influence could be the low variation in body weight found in this study.

It also remains unsettled why after the ossification is completed, at 45 wa, all hens ended up with a bone deviation or fracture, which seems to be a general phenomenon (7, 60). They experienced the same level of feather CORT deposition, regardless of the presence of damage. It is known that stress hormones like CORT in birds or cortisol in humans and other mammalian animals are important for the body's ability to respond to stress and injury. They are known to have complex effects on the skeleton, with small amounts needed for normal bone development but large amounts inhibiting bone growth (61). The finding of increased cumulative CORT deposition with age was similarly established in another recent chicken study (62). This in conjunction with the evidence in humans that prolonged treatment with glucocorticoids can produce osteoporosis (63), allowing us to speculate that our hens may produce osteoporosis characterized by a decrease in bone mass that may thus be related to KBD. In humans, it is reported (2) that bone adapts to stress with age, although its ability depends on both genetic factors and lifestyle, a phenomenon not yet proven in chickens. Nevertheless, knowing that the KB is typically reduced or absent in flightless birds (64, 65), likely as its main function is to provide adequate leverage for flight, and by assuming today's chickens are poor flyers (66) and very good egg producers, one can argue that the skeletal adaptation has changed with selection for high egg yield, increasing the frequency of KB breakage. Greater understanding of physiological and psychological stress-related relationships may help to reduce levels of damage and severity in modern chickens.

## Conclusions

Psychosocial factors such as fear-induced pecking and locomotor reactions and sociality revealed an association with the development of an adverse bone condition in hens. Knowing that an individual's success with surviving and reproducing depends critically on its behavior, in the present work, we propose that hens with poor bone condition may experience psychological consequences from KBD but also that fear- and social-related psychological stress may be a potential predictor of bone damage.

## Data Availability Statement

The raw data supporting the conclusions of this article will be made available by the authors, without undue reservation.

## Ethics Statement

All procedures with animals (experimental protocols and methods) were performed according to the legislation on animal experimentation in Slovenia and were approved by animal-welfare body at the Department of Animal Science, that is a member of Ethical Committee of the Administration of the Republic of Slovenia for Food Safety, Veterinary Sector and Plant Protection (UVHVVR) and were in accordance with the principals 3Rs.

## Author Contributions

NR contributed to the conceptualization, methodology, formal analysis, investigation, and writing the original draft. MZ contributed to the conceptualization, methodology, visualization, resources, writing, review and editing, and supervision. All authors contributed to the article and approved the submitted version.

## Conflict of Interest

The authors declare that the research was conducted in the absence of any commercial or financial relationships that could be construed as a potential conflict of interest.
